# Risk and Protective Factors for Cigarette Use in Young Adolescents in a School Setting: What Could Be Done Better?

**DOI:** 10.1371/journal.pone.0129628

**Published:** 2015-06-11

**Authors:** M. Dahlui, N. K. Jahan, H. A. Majid, M. Y. Jalaludin, L. Murray, M. Cantwell, T. T. Su, N. Al-Sadat, MyHeARTs group

**Affiliations:** 1 Centre for Population Health (CePH), Faculty of Medicine, University Malaya, Kuala Lumpur, Malaysia; 2 Department of Social and Preventive Medicine, Faculty of Medicine, University Malaya, Kuala Lumpur, Malaysia; 3 SEACO and School of Medicine and Health Sciences, Monash University Malaysia, Segamat, Malaysia; 4 Department of Paediatrics, Faculty of Medicine, University Malaya, Kuala Lumpur, Malaysia; 5 Centre for Public Health, Queen’s University Belfast, Belfast, Northern Ireland, United Kingdom; University of Florida, UNITED STATES

## Abstract

Smoking among Malaysian adolescents remains a public health concern despite concerted efforts in tobacco control. The aims of this study were to examine the prevalence and determinants of current-smoking status in young adolescents. This cross sectional study used the first round of the Malaysian Health and Adolescents Research Team’s prospective cohort study. It was conducted in three States of the Central and Northern regions of Peninsular Malaysia between March and May 2012. The study used the multistage stratified sampling design. A total of 1,342 adolescents of both sexes, aged 12-13 years, were sampled from randomly selected urban and rural national schools. Information on current smoking status and associated factors were collected by a self-administered, pre-tested, validated, structured questionnaire. Seven percent of the samples were current-smokers; the majority (62%) of them started smoking at the age of 11 years or below. The prevalence of current smoking was significantly higher in males (odds ratio [OR] = 2.37; 95% CI: 1.46, 3.84), those who were influenced by smoker friends (OR = 8.35; 95% CI: 4.90, 14.25), who were unaware of the health risks of smoking (OR =1.85; 95% CI: 1.02, 3.36) and who reported a lack of satisfaction about their overall life (OR =3.26; 95% CI: 1.73, 6.12). The study findings provide valuable information to strengthen the existing school-based smoking prevention program through integration of social competence and social influence curricula. The program should empower the young adolescents to refuse tobacco offers, to overcome social influences and to resist peer pressure to avoid starting smoking. Particular focuses to include mental health service to prevent both emotional and behavioural problems are needed.

## Introduction

Smoking is a well-established preventable risk factor for non-communicable diseases. While the prevalence of current tobacco use is declining among males aged 15 years and over globally (39% in 2005 and 36% in 2009) and in South-East Asia (39% in 2005 and 30% in 2009), current tobacco use is increasing in younger male adolescents aged 13–15 years globally (16% and 18% in 2007 and 2010 respectively) and in South-East Asia (17% and 21% in 2007 and 2010 respectively). In 2010, within the South-East Asian region, the prevalence of current smoking in young male adolescents (13–15 years old) was higher in Malaysia (35%) and Indonesia (41%) than that of the neighbouring countries (e.g. Philippines 28%, Thailand 24%, and Myanmar 23%) [1,2].

Behaviour-linked non-communicable diseases (NCDs), especially cardio-vascular diseases, diabetes and cancer etc., have become the leading causes of death in South-East Asia [3]. Malaysia has the highest number of overweight, obese as well as diabetic adults in the region [4], and has maintained an upward trend [5]. Hypertension, hyperglycemia, overweight and obesity are now also seen in Malaysian adolescents [6–9]. It is crucial for the future of the country and region that the major modifiable risk factors for NCDs, including smoking, are tackled within this particular group.

Hence, it is important to examine the determinants of smoking in early adolescence as the chances of remaining a regular smoker is high among those who start early (at or before 13 years of age) [10,11]. To date, the Malaysian studies that have examined the prevalence and determinants of tobacco use have mainly involved older adolescents (aged 16–17 years) and have been conducted at a local level [12–15]. An important exception is the recent school-based Malaysian national survey on smoking which included adolescents aged 12–13 years [16] but this study did not investigate the influence of peers, which has been identified as an important predictor of smoking among adolescents [17]. Therefore our study is aimed to investigate the prevalence and determinants of current smoking including the influence of peers among young adolescents. Such information is crucial in further strengthening of the existing smoking prevention interventions for Malaysian adolescents.

## Materials and Methods

This is a cross-sectional study. It uses the first round of data of the Malaysian Health and Adolescents Research Team study (MyHeARTs), which was a prospective cohort study with a plan to follow the study population until they become adults. The MyHeARTs study used a multistage stratified sampling design. At the initial stage, two (the Northern and Central regions) out of four educational regions of Peninsular Malaysia were selected based on the probability proportionate to the population size. At the second stage, a State in each region was selected, also with the probability proportional to the population size. Perak was selected in the Northern region with 10 administrative districts. Selangor having nine administrative districts with the Federal Territory of Kuala Lumpur (WPKL) were selected at the Central region.

In this study, the government schools were selected where the national language (Bahasa Malaysia) was the language of instruction. The schools were stratified into urban and rural based on the criteria provided by the Department of Statistics, Malaysia. The complete list of government secondary schools, which was collected from the Ministry of Education, Malaysia, was considered as the sampling frame.

At the third stage, government schools from both the urban and rural locations were randomly selected from the sampling frame by using computer-generated random number where each school had the same probability of being selected. In total there were 595 government schools (238 in Perak, 261 in Selangor and 96 in WPKL) which were stratified into urban and rural schools. Thus, 15 government schools (seven in Perak, five in Selangor and three in WPKL) were randomly selected. In the final stage, all eligible students within the selected schools were invited to participate in the study. Consent forms for both parents and students along with a detailed information sheet on the research project were distributed through school authorities. Finally, students eligible to participate in the study were those who submitted their completed written informed consent form along with the written informed consent form of their parents or guardians.

Based on the sample size calculation, the estimated sample size was 1,500 respondents. The sample size calculation was described in the MyHeARTs protocol paper [18]. There were 3,177 students from the 15 selected schools, 2,694 (85%) of whom were eligible for the study and received consent forms. Out of 2,694 students, 1,361 participated in the study giving an overall participation rate of 51%. This response rate varied by place of residence (66% in rural schools whereas 42% in urban schools) and by states (42% in Selangor, 50% in WPKL and 61% in Perak). Finally, 1,342 students (89% of the required sample size) completed the self-administered, pre-tested, validated, structured questionnaire.

The MyHeARTs researchers not only collected information on adolescent smoking behaviour, they also collected 15 mL fasting blood sample for different blood tests related to non-communicable diseases. Therefore, the participants had to fast for at least ten hours before blood draw on the next morning. This was the main reasons for low overall response rate. The detailed protocol of the MyHeARTs project is described in Hazreen et al. [18], in which the validation of the questionnaire was described. The study received ethical clearance from the Ethics Committee, University Malaya Medical Centre (Ethics Committee/IRB Ref. Number 896.34).

The study was undertaken between March and May 2012. The study population was male and female school children, aged 12–13 years, attending the first year of lower secondary school, and who were able to speak and write in the national language. Self-administered pre-tested validated structured questionnaires were used to collect the required information on current smoking status, socio-demographic characteristics, knowledge about harmful effect of smoking, overall satisfaction with life, communication with friends through social networking media, having smoker friends and smoker parents from the respondents who had given written consent to participate in the study.

## Statistical Analysis

The dependent variable examined was current smoking status, a dichotomous categorical variable, which was defined as smoking cigarettes daily i.e. at least one cigarette every day for the last 30 days. The main independent variables were peer influence and parental influence variables, which were created from the respective information of whether the respondents had any smoker friends (friends smoked or did not smoke) and smoker parents (either father only, mother only, or both.).

The study found that the respondents used several popular social networking media as a means of communication with friends. Those were: Facebook, Twitter, Myspace, Skype, and Yahoo Chat. Each of these five social networking media was an individual variable having two categories: communicated or did not communicate with friends. Finally, a composite variable was created from these five variables to create the “social networking media” variable.

Information on self-reported perception about overall satisfaction with life (whether the respondents were satisfied, dissatisfied or had mixed perception about life) and had knowledge about harmful effect of smoking were also considered as important predictors. Remaining potentially predisposing predictors were gender (male and female) as a demographic factor and place of residence (urban and rural) and ethnicity (Malay, Chinese, Indian and Others) as social structure.

Modelling: Associations between predictor variables and current-smoking status were examined using binary logistic regression. The relative odds of being a current-smoker compared to those who did not smoke were examined in three models to control for potential confounders. In the Model 1, the socio-demographic characteristics were included; Model 2 added the information of influence of peers, parents and social networking media and also the information on overall satisfaction with life as risk factors; and finally, Model 3 included the information on knowledge about harmful effect of smoking as a potential protective factor. The parameters of the logistic regression models were presented as adjusted odds ratios (OR), with 95% confidence intervals (CI). The significance value was set at p<0.05. Analysis was performed using the IBM SPSS software for Windows which was released in 2013 (Version 22.0. Armonk, NY: IBM Corp.)

## Results

The study mainly found that the likelihood of being a current smoker was significantly higher among those young adolescents who were male, were highly influenced by smoker friends and were unaware of the health risks of smoking and who reported a lack of satisfaction about their overall life. Table 1 presents the individual characteristics of the respondents (overall and by their current smoking status) and the findings of bivariate analysis.

**Table 1:** Factors associated with smoking status among respondents (N = 1342).

Predictor Variables		Total Sample Number	Current Smoker Number	Non-Smoker Number	P value
		%	%	%	
Gender	Male	513 (38.2%)	69 (69%)	444 (35.7%)	0.000
	Female	829 (61.8%)	31 (31%)	798 (64.3%)	
Ethnicity	Malay	1091 (81.3%)	84 (84%)	1007 (81.1%)	0.165
	Chinese	105 (7.8%)	3 (3%)	102 (8.2%)	
	Indian	105 (7.8%)	11 (11%)	94 (7.6%)	
	Others	41 (3.1%)	2 (2%)	39 (3.1%)	
Place of residence	Urban	714 (53.2%)	44 (44%)	670 (53.9%)	0.035
	Rural	628 (46.8%)	56 (56%)	572 (46.1%)	
Parental Influence	Parents smoke	644 (48%)	59 (59%)	585 (47.1%)	0.014
	Parents do not smoke	698 (52%)	41 (41%)	657 (52.9%)	
Peer Influence	Friends smoke	398 (29.7%)	77 (77%)	321 (25.8%)	0.000
	Friends do not smoke	944 (70.3%	23 (23%)	921 (74.2%)	
Knowledge about harmful effect of smoking	Have knowledge	1169 (87.1%)	79 (79%)	1090 (87.8%)	0.012
	Have no knowledge	173 (12.9%)	21 (21%)	152 (12.2%)	
Social Networking Media Influence	Communicate with friends using social media	979 (73%)	82 (82%)	897 (72.2%)	0.020
	Do not communicate with friends using social media	363 (27%)	18 (18%)	345 (27.8%)	
Overall satisfaction about life	Dissatisfied	105 (7.8%)	19 (19%)	86 (6.9%)	0.000
	Mixed satisfaction	144 (10.7%)	9 (9%)	135 (10.9%)	
	Satisfied	1093 (81.4%)	72 (72%)	1021 (82.2%)	
Total		1342 (100%)	100 (7.45%)	1242 (92.5%)	

Level of significance of *P* value: *P < 0.05; **P < 0.01; ***P < 0.001.

In this study, we observed that the majority of the respondents were female (61.8%) and Malay (81.3%). The respondents were almost equally distributed in the urban (53.2%) and rural areas (46.8%). Approximately half (48%) of the respondents had at least one parent (generally the father) who smoked. Slightly less than one-third (29.7%) of the respondents had friends who smoked and the majority (87.1%) knew the health disadvantages of smoking. The majority of the respondents (73%) reported communication with their friends through social networking media. Most of the respondents (81.4%) reported that they were satisfied with life.

Nine percent (120) of the students had ever tried smoking (Table 2) and 7.45% (100 students) were current-smokers (Table 3). More than half (62%) of the current smokers had started smoking at or below the age of 11 years (Table 4). The majority (69%) of the current smokers were male; more than two-third (77%) had smoker friends and slightly more than half of them (59%) had smoker parents. Table 1 also presented the results of bivariate analysis where we had found significant relationships between current-smoking status and all proposed predictors, except ethnicity.

**Table 2:** Frequency distribution of the study respondents who have ever tried or experimented with cigarette smoking, even one or two puffs.

Smoking Characteristics	Frequency	Percent
Ever smoker	120	8.9
Never smoker	1222	91.1
Total	1342	100.0

**Table 3:** Frequency distribution of the study respondents who were current smokers and Non-Current smokers.

Smoking Characteristics	Frequency	Percent
Current smoker	100	7.45
Non-Current smoker	1242	92.55
Total	1342	100.0

**Table 4:** Frequency distribution of the study respondents by their age of smoking initiation.

Age of smoking initiation	Frequency	Percent	Cumulative Percentage
7 years old or younger	12	12	12
Within 8 to 9 years	23	23	35
Within 10 to 11 years	27	27	62
12 years or above	38	38	100
Total	100	100	

The associations with gender, peer influence, overall satisfaction about life, and knowledge about the health disadvantages of smoking persisted in the multivariate analysis after controlling the potential confounders. In Table 5, the findings are presented in three models to control potential confounders. In Model 1, the socio-demographic characteristics were included; in Model 2 we controlled individually the information of influence of peers, parents and social networking media and also the information on overall satisfaction with life as risk factors; and finally, in Model 3 we controlled the information on knowledge about harmful effect of smoking as a potential protective factor.

**Table 5:** Adjusted Odds Ratio for factors affecting current smoking behaviour.

Explanatory Variables		Model 1	Model 2	Model 3
		OR (95% CI)	OR (95% CI)	OR (95% CI)
Gender	Female^#^			
	Male	3.97***	2.47***	2.37***
		(2.55 to 6.19)	(1.53 to 4.00)	(1.46 to 3.84)
Ethnicity	Malay^#^			
	Chinese	0.39	0.47	0.41
		(0.11 to 1.31)	(0.13 to 1.66)	(0.11 to 1.48)
	Indian	1.74	3.71**	3.18**
		(0.86 to 3.53)	(1.65 to 8.33)	(1.39 to 7.29)
	Others	0.58	0.94	1.04
		(0.13 to 2.51)	(0.20 to 4.35)	(0.22 to 4.82)
Place of residence	Rural^#^			
	Urban	0.78	0.70	0.71
		(0.50 to 1.21)	(0.43 to 1.13)	(0.44 to 1.16)
Peer influence—having friends who smoke (Yes/No)	Yes^#^			
	No		8.32***	8.35***
			(4.88 to 14.19)	(4.90 to 14.25)
Parental influence—parents who smoke (No/Yes)	No^#^			
			1.03	1.07
	Yes		(0.65 to 1.64))	(0.67 to 1.70)
Social networking and media influence-communicate with friends using social media	No^#^			
	Yes		1.54	1.61
			(0.84 to 2.82))	(0.88 to 2.96)
Overall satisfaction about life	Satisfied^#^			
	Dissatisfied		3.32***	3.26***
			(1.77 to 6.24)	(1.73 to 6.12)
	Mixed Satisfaction		1.06	1.03
			(0.49 to 2.27)	(0.47 to 2.22)
Knowledge about harmful effect of smoking (Yes/No)	Yes^#^			
	No			1.85*
				(1.02 to 3.36)

Level of significance of *P* value:

*P < 0.05;

**P < 0.01;

***P < 0.001.

^#^ indicates the reference category.

In Model 1, a significant relationship was found between current-smoking status and male gender (odds ratio [OR] = 3.97; 95% confidence interval [CI]: 2.55 to 6.19, p <0.001). This relationship remains significant even after controlling for other predictor variables in Models 2 and 3. In Model 2, an overall significant effect (Wald statistics = 12.62, df = 3, p<0.01) of ethnicity was found on the current smoking status. Indian respondents were significantly more likely (OR = 3.71 95% CI: 1.65 to 8.33, p <0.01) to become current-smoker than Malay after controlling for other risk factors. Peer influence was found strongly related to the odds of being a current-smoker (OR = 8.32 95% CI: 4.88 to 14.19, p <0.001); the likelihood of being a current-smoker was eight times higher among those respondents whose friends smoked, compared to those whose friends did not smoke.

The overall effect of satisfaction about life (Wald statistics = 14.24, df = 2, p<0.01) was also significant. A strong statistically significant relationship was found between current-smoking status and overall satisfaction about life; respondents who reported that they were not satisfied with life three times as likely to be smokers (OR = 3.32; 95% CI: 1.77 to 6.24, p <0.001) compared to those satisfied with life. All these relationships remained unchanged even after controlling the knowledge on the harmful effects of smoking as a protective factor in Model 3. In this final model, an association existed between current-smoking and reported lack of knowledge about the harmful effects of smoking (OR = 1.85; 95% CI: 1.02 to 3.36, p <0.05); those who were unaware of the health risks of smoking were about 85% more likely to be a current-smoker. Besides, in the full model, the likelihood of being a current-smoker remained higher among those who were male (OR = 2.37; 95% CI: 1.46 to 3.84, p <0.001), Indian (OR = 3.18; 95% CI: 1.39 to 7.29, p <0.01), had friends who smoked (OR = 8.35; 95% CI: 4.90 to 14.25, p <0.001), and perceived a lack of satisfaction about their overall life (OR 3.26; 95% CI: 1.73 to 6.12, p <0.001). Parental smoking, use of social media, and place of residence were not associated with smoking in the final model.

## Discussion

Both this study and the Malaysian nationwide school-based tobacco survey [16] found the same prevalence of current smoking (7%) among young adolescents aged 12–13, and both studies showed an early age of smoking initiation. These findings highlight the vulnerability of Malaysian younger adolescents to tobacco dependence. Evidence shows that young adolescents who are exposed to nicotine become addicted within a very short period. The possible cause is due to the immaturity of their brain allowing nicotine to have more disruptive effects on brain function during this critical period of growth and development [19,20].

Consistent with existing literature [12–14], this study also found that adolescent boys are significantly more likely to be smokers than girls; hence smoking prevention programmes mainly need to focus on young male adolescents. In Malaysia, smoking is still common among men but smoking prevalence is increasing in women, especially young women [21]. In our study almost four percent of the girls smoked, compared to 13% of boys and almost a third of current smokers were girls. It is possible that girls underreported smoking as it is not a conventional societal custom for Malaysian women. However, this study’s finding related to female smoking is significantly higher than the prevalence (around 0.5%) reported in adult females in the latest national health and morbidity survey [5] and draws due attention.

We also observed an association between current-smoking and ethnic status, after controlling for other risk factors. The Indian young adolescents were more likely to be smokers than the Malays, which is not consistent with the previous study among adult population [22], but is consistent with the national Malaysian survey conducted among older people aged 50 years, in which they found that compared to Malay, Indian older generation significantly suffered from coronary heart disease where smoking was a known risk factor [23]. Risk of being a smoker was low among our Chinese respondents, similar to another Malaysian study which found that smoking cessation was most common among Chinese adults due to their concerns related to health [24]. It is possible that Chinese adolescents in this study had received similar health related message from their elders which discouraged them to adopt this risky health behaviour compared to other ethnic groups in Malaysia.

Our study shows a strong association between peer influence (having friends who smoke) and being a current smoker. This finding is consistent with many previous studies [12–15,25,26] where peer influence has been shown to be one of the most important risk factors for smoking, including influencing the transition from an experimental to a regular smoker [27]. We did not find any association of parental smoking with the smoking status of our respondents, which suggests that Malaysian adolescents are more strongly influenced by the behaviour of their friends in the school environment, than their family or home environment. Although this finding is not consistent with the previous studies [28–30], it complies with the ‘Problem-Behaviour Theory’ [31] where peer influence is considered to have a direct effect on smoking and parental influence to be less important, and with the ‘Social Learning Theory’ [32] where school environment plays an important role in adolescents observing and adapt risky behaviours.

In our study, the bivariate association between communication with friends through social media and current smoking status was attenuated in the multivariate model. Communication with friends through social networking media is common among teenagers, including in this study. Use of these media may not influence smoking patterns in adolescents over and above other means of communication within this group. In a recent US study [33] use of social media was not found to be associated with the adoption of hazardous behaviours among adolescents unless the social media were used in a precarious way, such as sharing pictures of their smoking with friends.

We observed a strong association with current-smoking status and self-reported dissatisfaction with overall life. Self-reported overall life dissatisfaction and depression are correlated [34], and evidence exists to show that adolescents start smoking when they perceive that they are depressed or feeling stressed [14,35], and this may be the case among smokers in this study. Information on the reasons for dissatisfaction with life will be collected during the follow-up phases of this longitudinal study. It is important to understand the reasons for dissatisfaction with life at this young age as effective interventions can prevent depression as well as smoking in early adolescence [36,37]. Evidence found that school-based mental health program, especially in the US, has a strong impact on prevention of emotional (the most common is depression) and behavioural problems [38]. Another study also suggested to involve paediatricians, mental health specialist together with educators to improve the effectiveness of school-based mental health service [39]. Hence, we recommend that mental health components should be included in the school based smoking prevention program in Malaysia.

The majority of the respondents of this study knew that smoking is harmful to health., This may be due to the presence of graphic pictorial messages on cigarette packets. Government media campaigns also played an important role to increase knowledge. As may be expected, lack of awareness of the health effects of smoking was associated with an increased risk of smoking, which demonstrates the importance of ensuring that messages on the detrimental health effects of tobacco reach all sections of the community, including the very young.

The Malaysian government has undertaken wide-ranging nationwide activities to control tobacco use. Under the legislation of “Control of Tobacco Product’ Regulations in 1993”, smoking is forbidden in public and private places including educational institutions; all forms of tobacco advertisement and promotion are banned; pictorial health warning messages on cigarette package have been introduced, and tax, import and excise duties on cigarettes increased. Since 2004, it has been illegal for anyone aged below 18 years to purchase cigarettes and the sale of single cigarettes or small packs (14 cigarettes per pack) is also legally prohibited. Also, a nationwide anti-smoking media campaign “Tak Nak Merokok (Say No to Smoking)” was implemented between 2004 and 2011 to increase awareness of the health effects of smoking both at individual and societal levels [40]. Since 1991, “A Healthy Lifestyle Campaign” was also implemented by the government with emphasis on five major components related to healthy lifestyle including smoking. Smoking cessation service through “Quit Smoking Clinic” is also available throughout the country [41], but the study found that additional efforts are needed for the effectiveness of these clinics [42]. Hence, smoking still remains a public health concern. This shows the importance of having more concerted efforts especially on those who had never smoked from becoming smokers due to the fact that when one starts smoking, it is very difficult to quit.

Since children and adolescents spend most of their time at schools, the school setting could be considered as one of the best platforms to implement intervention programs for young generation. Keeping it in mind, the Malaysian government has taken several school based initiatives in combating smoking habit. Since 2007, “Program Doktor Muda (Young Doctors Programme) was integrated into the co-curricular activities of primary schools under the Ministry of Education to empower the primary school children with knowledge and skills on healthy lifestyles through peers. “PROSTAR- Program Sihat Untuk Remaja (Health Programme for Teens) is another school-based program that was initiated in 1990s also focused on empowerment of adolescents through peer educators on a wide range of topics such as risky behaviour, sexual and reproductive health and HIV/AIDS, as well as physical and environmental health [37]. Even though these programs have been in force for many years, smoking amongst young Malaysian adolescents still exists.

The recent Systematic Review found that school based smoking prevention interventions were effective in preventing young students from starting smoking if those interventions were implemented by adults, for long term (one year and more), and incorporated both social competence and social influences curricula. Thus, the interventions help the young people to be socially competent to refuse tobacco offer; to overcome social influences and to deal with peer pressure to avoid starting smoking compare to those programs which focused on only to provide information [43].

Peer-led school based smoking prevention was also found to be effective. For example, the ASSIST (A Stop Smoking in Schools Trial), a peer-led intervention with 12–13 years adolescents in 59 schools of England and Wales was found effective to increase children’s decision-making skills and self-esteem and to improve their ability to resist social and peer influences to smoke [44]. This programme has also been shown to be cost-effective [45].

This study found peer influence was the most important determinant of smoking initiation; hence it is crucial to develop the social skill of the Malaysian adolescents to resist peer pressure and to refuse offer to smoke. At the same time, the potential of peer pressure in influencing healthy behaviours could be exploited. This potential has actually been taken up when the Ministry of Health launched on the Project Rakan Muda (Young Friends/Peers Project) for the health education on HIV/AIDs prevention at schools. It is timely that the existing Malaysian school based smoking prevention program should be strengthened through incorporation of the social competence and social influence curricula. Initiatives are needed to incorporate the social skills for adolescents to resist peer pressure of smoking and other risky behaviours, in the educational curriculum. A particular focus should be given to include mental health service in preventing both emotional and behavioural problems. A long term follow-up is needed for the effectiveness of the interventions. Such empowerment and skills could help to prevent these adolescents from experimenting and taking up any risky behaviour and could also in a way assist them in coping with mental issues. This study finding generates evidence not only for Malaysia, but also for the neighbouring countries with similar problems.

We acknowledge that there were several limitations in this study. Allowing the students to decide on participation and the reliance on self-reported smoking status of the respondents could have underestimated the findings. The low response rate was because this study was a part of the prospective cohort study which involved blood taking. We should have asked the non-attendees who had consented to participate to answer the questionnaires on smoking; which was not possible since the granted period of data collection at the schools was very short. Nevertheless, a low response rate among adolescents is not uncommon and a cross sectional study had found that there were no differences in the characteristics between participating and non-participating children [46].

## Conclusion

Despite having some limitations, our study provides crucial information to the government. To reduce smoking prevalence and to assist the Malaysian government to achieve the mission of having a smoke-free country by 2020, it is essential to prevent smoking initiation in early adolescence and provide them with the skills to resist peer influence on risky behaviours. Therefore, further investment is needed to strengthen the existing school based interventions to prevent young adolescents from initiating or continuing to smoke.
